# Retrospective analysis of the efficacy of radio-contrast-induced nephropathy prophylaxis

**DOI:** 10.1186/cc14376

**Published:** 2015-03-16

**Authors:** J Wood, N Shields, KV Wood

**Affiliations:** 1 Maidstone & Tunbridge Wells NHS Trust, Maidstone, UK

## Introduction

This study investigated renal outcomes following radio-contrast (RC) administration in patients from two intensive care units (ITUs), where one gave RC-induced nephropathy (RCIN) prophylaxis, while the other did not. Acute kidney injury (AKI) during critical illness increases morbidity and mortality. ITU patients, who already suffer a variety of renal insults, often require RC, increasing their risk of developing AKI, and requiring renal replacement therapy (RRT). Evidence suggests that hydration alone is inadequate for the prevention of RCIN in ITU patients, and is contraindicated in some disease states [1]. The European Society of Intensive Care Medicine (ESICM) provides recommendations for prophylaxis [2]. The current study aimed to establish the efficacy of the ESICM recommended prophylaxis.

## Methods

Retrospective data from 140 Maidstone (M) ITU patients (men 101, women 39, mean age 63.5, mean APACHE 15.3) and 73 Tunbridge Wells (T) ITU patients (men 41, women 32, mean age 60.2, mean APACHE 20.2) admitted between 22 September 2011 and 22 September 2013, who underwent RC-enhanced CT, were collected. Patients on MITU received ESICM-recommended RCIN prophylaxis: 200 mg aminophylline i.v. over 30 minutes prior to RC, 1.26% sodium bicarbonate 3 ml/kg/hour for 1 hour prior to RC and 1 ml/kg/hour for 6 hours post RC. TITU patients received standard critical care alone. Exclusion criteria were: those undergoing RRT prior to CT, >1 CT in 48 hours, no creatinine (Cr) data available post scan. The Cr prior to CT (baseline), at 24, 48 and 72 hours post CT scan were identified. The RIFLE criteria was used to classify the changes of Cr from baseline into low risk (% change >1.25), risk (% change >1.5), injury (% change >2) or failure (% change >3, or Cr>355 and increase of >44).

## Results

The total number of patients developing renal injury falling into any RIFLE category for MITU at 24, 28 and 72 hours was eight (0.06%), 12 (0.09%) and 14 (0.1%), while for TITU it was five (0.07%), six (0.08%), and four (0.05%) respectively. A repeated-measures ANOVA revealed no significant differences in outcomes between the two groups overall (*F *= 2.35, *P *= 0.127) or at each time point (*F *= 1.93, *P *= 0.123).

## Conclusion

While RCIN is a recognised problem within the critical care population, there is little clear evidence for any prophylactic strategy to reduce this risk. This study suggests that a RCIN prophylaxis protocol based on the ESICM recommendations has no effect on the incidence of RCIN. However, further studies are needed.
